# Imitation in autism: why action kinematics matter

**DOI:** 10.3389/fnint.2012.00117

**Published:** 2012-12-13

**Authors:** Emma Gowen

**Affiliations:** Faculty of Life Sciences, The University of ManchesterManchester, UK

## Introduction

Humans have a flexible approach to imitation. If an action has a visual goal or is meaningful, we will “emulate” that goal using the most familiar or comfortable response from our existing motor repertoire. However, if the action is meaningless or lacks a visual goal we will more closely imitate the kinematic details of the action such as its amplitude, speed, or trajectory (Bekkering et al., 2000; Rumiati and Tessari, 2002; Carpenter et al., 2005; Wild et al., 2010). This pattern can be explained by two theories. The goal-directed theory of imitation (GOADI, Bekkering et al., 2000; Wohlschlager et al., 2003) suggests that during imitation, the observer cognitively decomposes the observed action into a hierarchy of goals, based on functionality: the visual goal of the action (pointing to a dot) is given more importance than the means (which hand to point with), causing the goal to be imitated rather than the means. In the absence of a visual goal, the movement itself moves up the hierarchy to become a primary goal and is preferentially imitated. The dual-route model of imitation (Rumiati and Tessari, 2002) proposes that for imitation of unfamiliar actions there is a direct mapping of the visual information onto a motor response, and for the imitation of known, meaningful actions, there is an indirect, semantic route which utilizes long term memory. Both models suggest that when an action lacks either meaning or a visual goal, imitation will reflect the observed movement more closely due to greater attention to, and visuomotor mapping of, the movement rather than the visual goal.

In contrast, autistic people do not show this flexible approach to imitation. Autistic children often display similar performance to neurotypical children when imitating actions that have a visual goal or meaning but are less able to imitate goal-less or meaningless actions (Rogers et al., 1996, 2010; Stone et al., 1997; Hobson and Lee, 1999; Williams et al., 2004; Hamilton et al., 2007a; Vanvuchelen et al., 2007; Hobson and Hobson, 2008; Cossu et al., 2012). We recently demonstrated a similar pattern for the first time in autistic adults (Wild et al., 2012). Participants observed, then imitated videos of a hand making two movements while their own hand and eye movements were recorded. In the goal-directed condition, the hand moved between visual targets and in the goal-less condition the hand made similar size movements without any visual targets. The hand in the video moved at either a fast or slow speed in order to determine whether participants modulated their imitation speed accordingly. In line with GOADI, neurotypical participants imitated speed changes in the goal-less but not the goal-directed condition whereas the autistic participants used a goal-directed approach, failing to modulate their imitation speed across conditions. In addition, eye movement data indicated that the neurotypical participants spent more time attending to the hand, particularly during the goal-less condition whereas the autistic participants attended to the visual targets and hand equally across conditions.

From the above evidence, it is apparent that when successful imitation requires attending to and using kinematics, autistic performance is particularly affected. In the following, I will highlight the functional significance of this pattern by suggesting that autistic people have a bias away from observing and analysing kinematics, which results in a significant loss of social information. I will outline three behaviors where attending to and imitating kinematics is important for social interaction and discuss the impact for autistic people if their ability to use kinematics is compromised.

## Kinematics and prediction

Observing kinematics helps us to understand and predict the actions of others (Shim and Carlton, 1997; Pozzo et al., 2006; Graf et al., 2007; Hamilton et al., 2007b; Aglioti et al., 2008; Ambrosini et al., 2011; Becchio et al., 2012; Stapel et al., 2012). For example, by observing the initial portion of an action, people are able to tell whether an actor is deceiving them about the weight of a lifted box (Grézes et al., 2004) or whether a reach-to-grasp action is performed under a cooperative or competitive situation (Manera et al., 2011). Moreover, when observing an action where there are multiple targets, we are able to use kinematic information from the shaping of the hand to correctly identify the appropriate target (Ambrosini et al., 2011; Paulus et al., 2011). If autistic people do not use kinematic cues, one would expect them to perform poorly on similar action prediction tasks. For example, they may find it hard judging the end point of an action or detecting behavioral changes in other people such as a physical illness (e.g., a motor disability), leading to misreading of social situations, confusion, and altered social responses to other people. Although autistic performance on action prediction tasks requires testing, some evidence does point to difficulties using kinematics. Boria et al. (2009) asked participants to decide why an action was being performed. The action could either be congruent with the functional use of the object (e.g., picking up the receiver of a phone) or unconventional (e.g., picking up the phone using a grip suggesting the actor is intending to move it). Autistic children performed worse then neurotypical controls only in the unconventional conditions, suggesting that they were weighting the functional use of the object over the hand action (see also Hammes and Langdell, 1981; Cossu et al., 2012). Furthermore, in contrast to neurotypical children who imitate intentional actions more frequently then accidental actions, autistic children were found to imitate both types equally (D'Entremont and Yazbek, 2007). As accidental actions were differentiated from intentional actions by both a verbal “whoops” and different (e.g., jerkier) kinematics this suggests that the autistic children were unable to use these cues to detect the intentional action.

## Kinematics and learning

As kinematics provide knowledge about the purpose of an action, it follows that if we fail to comprehend the goal of an action we can attend to, and imitate, the kinematics in order to more fully understand that action. Indeed, both kinematics and knowledge of the goal are important when learning new actions through observation and imitation (Hayes et al., 2007, 2008) and imitation via the direct, visuomotor route is more effective for learning then the indirect route (Rumiati et al., 2009). In addition, Williamson and Markman (2006) demonstrated that compared to situations where there was a clear purpose to the modeled action, children reproduced the action more faithfully if there was no clear reason for the model to perform that action. Consequently, if observational learning relies to some extent on direct visuomotor mapping one might expect that learning novel actions would be harder for autistic people. It is possible that they would learn better by doing it themselves first in order to acquire the motor representation and perhaps rely more on proprioceptive rather than visual information to learn (Haswell et al., 2009). Little work has examined how well-autistic people learn via observation and imitation, but it was recently observed that compared to neurotypicals, autistic children required additional demonstration and practice to learn how to retrieve a prize from a custom built box (Nadel et al., 2011). However, more experiments are required to fully test this form of learning, particularly using tasks where success depends on learning kinematics (e.g., retrieving the prize required a certain movement speed or trajectory).

## Kinematics and social response

Observing kinematics allows us to predict other people's actions, but also provides information about how to respond to others—e.g., whether we should imitate to learn, play or “fit in.” This social function of imitation is apparent in situations where children and adults imitate unnecessary or unusual actions (Gergely et al., 2002; Whiten et al., 2009; McGuigan et al., 2011). For example, when asked to retrieve a reward from a box, participants imitate causally irrelevant actions that clearly have no impact on the success of retrieving the reward (McGuigan et al., 2011). However, when there is an apparent reason for the irrelevant action (e.g., an accident), infants are more likely to imitate only the goal (Meltzoff, 1995; Carpenter et al., 1998; D'Entremont and Yazbek, 2007). It has been suggested that this form of imitation serves a social role, providing a shared experience and a way to conform and align oneself with ones cultural group (McGuigan et al., 2011; Nielsen and Blank, 2011; Simpson and Riggs, 2011). Depending on the context, we may interpret the unusual kinematics of an action as an invitation to join and share the experience (Rogers et al., 2010) to learn or to conform. I suggest that this behavior stems from a comparison between the (known) goal and the unusual kinematics of the action, resulting in a prediction error and alerting the observer to pay more attention to the action. Importantly, kinematics signal that the action requires re-evaluation and that it may be appropriate to imitate the action more closely to play, learn, or conform. In line with a failure to use this kinematic information, autistic, compared to neurotypical children are less likely to imitate actions that do not have a clear function or are incidental to achieving the outcome (Hobson and Hobson, 2008; Rogers et al., 2010; although see Nielsen et al., 2012). We also found a similar pattern in adults carrying out imitation of hand movements when the observed hand made a curved movement instead of moving straight to the end location (Wild et al., 2012). Neurotypical adults imitated the curved trajectory in both the presence and absence of visual goals, whereas the autistic adults only imitated the trajectory in the absence of goals. These results suggest that in the presence of a clear visual goal, neurotypical participants place significance on the unusual movement trajectory by analyzing the kinematics and changing imitation strategy, whereas the autistic participants weighted the visual goal.

## Concluding remarks

I have highlighted how the pattern of imitation impairments in autism can provide a key to understanding autistic behavior. Autistic individuals have greater difficulty imitating actions that require close observation and visuomotor mapping of kinematics, suggesting that they are failing to use kinematics to predict, learn, or respond appropriately. Consequently, they are missing out on a rich source of social information. Future work is required to directly test how autistic individuals perform action prediction and observational learning tasks in order to advance this theory. It is also important to find out why autistic individuals are less inclined to use kinematic information. Although a number of studies have found that motor difficulties cannot solely account for imitation impairments (Rogers et al., 1996, 2003, 2010; Dewey et al., 2007; Vanvuchelen et al., 2007; Wild et al., 2012) it is arguable that observing and imitating kinematics places particular demands on visuomotor control (Press and Heyes, 2008; Rumiati et al., 2009). As biological motion is dynamic and fast it may be relatively more challenging for autistic people to integrate visual with motor signals, compared with standard motor test batteries that often require self-generated movements. Alternatively, our previous eye tracking results suggest a reduction in attention toward the kinematics in favor of the goal, potentially due to altered top down control (Wild et al., 2012). Importantly, this does not imply a reduction of general attention to the task (Press et al., 2010), but a specific bias away from the kinematics. Altered attention is consistent with theories proposing that autistic people fail to attend to social stimuli because they do not experience feelings of social reward (Dawson et al., 2004; Chevallier et al., 2012). Consequently, autistic individuals may feel little motivation to attend to and imitate the kinematics, which contain socially relevant information. Whether the failure to use kinematics is due to visuomotor impairment or altered attention is important as it affects how we may design future training therapies. It will be critical to test whether training can enable autistic people to successfully attend to and imitate kinematics and whether this results in improvements in prediction, learning, and social response.
